# Supplementary material for: Are carbon emissions trading and green financial instruments synergistic? -Comprehensive quantitative research based on content analysis
Source: PLoS One. 2024 Mar 7;19(3):e0298601. doi: 10.1371/journal.pone.0298601 (PMC10919601; doi:10.1371/journal.pone.0298601)
Supplement: S1 Data — (ZIP) [file pone.0298601.s001.zip › Data/地级市Z_F工作报告.zip!/║■─╧/│ñ╔│╩╨/└┤╫╘╣1⁄2╓┌║┼í╛┬φ┐╦╩2╛▌═°í┐_2018.docx]

各位代表：

　　现在，我代表市人民政府向大会报告工作，请予审议，并请各位政协委员和其他列席人员提出意见。

　　一、2017年工作回顾

　　2017年，我们在省委、省政府和市委的坚强领导下，喜迎党的十九大，宣传贯彻落实十九大精神，不忘初心，砥砺奋进，大力实施“创新引领、开放崛起”战略，加快打造国家智能制造中心、国家创新创意中心、国家交通物流中心，完成了市十五届人大一次会议确定的目标任务。全市地区生产总值10200亿元（预计数，下同），增长9.0%；规模工业增加值3540亿元，增长8.5%；固定资产投资7570亿元，增长13.1%；社会消费品零售总额4550亿元，增长10.5%；一般公共预算收入1400亿元，增长13.73%。经济社会发展实现稳中求进、进中向好。

　　——经济总量迈入“万亿俱乐部”，长沙站上了新起点。

　　——岳麓山国家大学科技城、马栏山视频文创产业园、临空经济示范区、高铁会展新城、湖南金融中心等重大片区建设精彩开局，南部片区开展了规划编制。

　　——工业30条、人才新政22条、科技创新“1+4”和开放型经济“2+4”等政策叠加实施、落地见效，汇聚了蓬勃动力。

　　——国家临空经济示范区获批，黄花综合保税区封关运行；首条定期国际全货机航线开通；老挝总领事馆设立，实现全省领事机构零的突破。

　　——新增上市企业12家，累计达66家，占全省的75.59%，A股上市企业数量和市值均居中部首位。

　　——东亚文化之都活动精彩纷呈；被联合国教科文组织评为世界“媒体艺术之都”，是全国第一个获此殊荣的城市。

　　——精准扶贫、精准脱贫成效明显，68个省定贫困村全部退出，6.4万贫困人口全部达到脱贫标准，率先建成全面小康社会。

　　——打赢抗洪救灾保卫战，长沙经受住湘江39.51米创纪录洪峰水位的严峻考验，谱写了战天斗地的抗洪壮歌。

　　一年来，我们始终坚持以人民为中心的发展思想，践行新发展理念，严格落实从严治党主体责任，把权力关进制度的笼子，自觉接受市人大及其常委会的法律监督、工作监督和市政协的民主监督，以政府改革带动全面改革，以制度创新带动全领域创新，以增强执行力提升行政效能，打造政策落地的“长沙速度”，兑现政府承诺，政府公信力明显增强。着力抓了七个方面的工作：

　　一是着力发展产业园区，促进转型创新升级。湖南湘江新区发展走在全国新区前列，示范引领、辐射带动作用持续增强。恒大童世界、华谊电影小镇、湘江欢乐城、湘军文化园等一批重大项目加速推进。园区特色更加彰显，国家级园区“两主一特”、省级园区“一主一特”产业功能明晰，长沙经开区汽车及零部件产业产值过千亿元，全市每两分钟不到就有一辆新车下线；浏阳高新区获批中国产学研合作创新示范基地，岳麓科技产业园评为国家检验检测认证公共服务平台示范区，隆平高科智慧园区建设富有成效，国家文化产业园、广告产业园合并获批全国版权示范园区，全市规模以上工业增加值园区占比达63.6%，提升2.5个百分点。项目建设成效显著，全市932个重大项目完成投资4200亿元，中信戴卡一期、特格尔、比亚迪电动卡车及专用车等项目建成投产，中国通号全球首台智能电力有轨电车上市，智能系统测试区、湖南地理空间大数据应用中心建成；蓝思3D、新科电子、海尔无线、长利智能家居、中民筑友、中南大学科技园研发总部等加快推进；新引进五矿新能源、广汽三菱发动机、中国电科等投资50亿元以上项目23个，特别是第六代柔性面板、华大基因、伟创力、铁建重工、中国长城等一批百亿元级项目相继落户，长沙智能制造发展进入新阶段。创新驱动引领转型发展，万人有效发明专利拥有量居省会城市第5位，新认定高新技术企业800多家，增幅居中部首位，高新技术产业总产值突破万亿元、增加值占地区生产总值31%，“麓谷创新指数”持续攀升；湖南省军民融合科技创新产业园、长沙军民融合双创示范园加快建设，北斗产业安全技术研究院协同创新作用凸显；工程机械、新材料、电子信息、食品、生物医药等支柱产业优势进一步巩固，移动互联网、3D打印等产业产值保持50%以上增幅。平台建设加快推进，新增航天中南先进光机电技术创新中心、湖南大学机器人研究院等研发机构，新建院士专家工作站9家。国家智能制造示范企业及专项总量达20个，稳居中部第一；长沙工业云注册企业达1600家并向全省覆盖，“树根互联”工业互联网平台成为产业革命风向标。工业对经济增长贡献率提升5个百分点。服务业持续发展，经济增长贡献率超过50%。全域旅游加快推进；湘江基金小镇挂牌成立；红星全球农产品批发中心一期建成；智能制造、移动互联网等峰会成功举办，国际轨道交通、工程机械配套件博览会以及筑博会、农博会、食博会等会展蓬勃发展。

　　二是着力深化改革开放，持续增强发展活力。28项“国字号”改革试点有序实施，84项改革纵深推进。“放管服”改革持续深化，开展“减证便民”专项行动，取消各类证明145项。赋予湖南湘江新区市级权限44项，向省级以上园区下放权限89项；湖南湘江新区率先试点产业用地管理改革，实现投资审批“一网通办、在线办结”；芙蓉区成立全省首家行政审批局，实现“一枚印章管审批”；浏阳发布1012项“最多跑一次”事项；宁乡市民服务中心达到国家政务服务综合标准。深化商事制度改革，全面推行电子营业执照、企业登记便利化和“43证合一”，企业登记审批时限缩短91%，新增商事主体18.3万户，每万人市场主体达1051个，居中部省会城市首位。深化供给侧结构性改革，“1+7”政策落地见效，全市为企业及职工降低社会保险费10.45亿元，“营改增”减税194亿元；限购限售、“限房价+竞地价+摇号”、公积金贷款调整等调控政策精准发力，房地产市场持续健康稳定发展；农村土地“三权分置”、扩权强镇等改革成效明显，望城、宁乡成为全国农村改革试验区，雨花区开展农村集体产权制度改革全国试点。深化其他领域改革，宁乡撤县改市；试点国企混改，组建建发集团；探索形成“土地托管+基层社”供销合作社改革新模式；完成知识产权综合管理改革，实现专利权、商标权、著作权“三权合一”。对外开放步伐加快，新增白俄罗斯莫吉廖夫市、荷兰莱瓦顿市等5个友好城市，与194个国家（地区）实现贸易往来，完成进出口总额130亿美元，增长18%。“湘粤港直通快车”开通，进境食用水生生物等3个指定口岸建成或通过验收，新开国际及地区航线14条，获批开展保税航油业务，中欧班列（长沙）货运量全国第四；16个国家在长设立签证申请中心，驻长世界500强企业达151家；成功举办泛珠三角区域省会城市市长联席会，承办泛珠三角区域合作行政首长联席会议、第十五届东亚论坛、金砖国家协调人第五次会议、第十一届中国企业跨国投资研讨会，开放崛起的长沙越来越聚焦世界目光。

　　三是着力改善环境质量，推进生态文明建设。中央环保督察1503件信访交办件基本销号，省政府交办的31个突出环境问题按期办结，解决了浏阳河城区段积淤等重大环境问题。全面推行“河长制”，深入实施湘江保护与治理“一号重点工程”，全面禁止环保不达标船舶进入湘江，加快浏阳河、沩水沿线畜禽养殖退出，新改扩建污水处理厂6个；集中式饮用水水源地水质达标率100%，建成圭塘河生态引水工程，成功创建全国首批水生态文明城市。持续推进大气污染防治，淘汰黄标车29497辆，新增纯电动公交车1609辆，完成居民家庭餐厨油烟净化治理改造2万户，建筑工地扬尘在线监测实现全覆盖，全年空气质量优良天数263天，优良率达72%。铬盐厂土壤污染治理有序推进。造林1万公顷，森林覆盖率、绿化覆盖率分别达54.82%、41.5%。率先推广两型标准认证、实施系统生态补偿，综合配套改革经验全国推介，获批全国低碳试点城市，两型社会建设成为靓丽名片。

　　四是着力提标提质提档，加快建设品质长沙。强化规划引领，城市总规修编、城市“双修”等国家改革试点深入开展，率先全国实现控规层面城市设计全覆盖；“多规合一”平台试运行。完善城市功能，建筑设计、市政工程等行业标准全面提升；长株潭城际铁路全线通车，星沙联络线、机场大道建设及金星路、潇湘大道、福元西路等提质改造完成，地铁3－6号线、湘府路快速化、西二环拓改和49条断头路、瓶颈路建设有序推进；建成自行车道408公里、步行道646公里，“公交都市”加快创建；市四医院等建设进展顺利，黑麋峰垃圾焚烧项目竣工，市青少年宫试运营，市妇女之家启用；完成棚改17466户190.56万平方米，改造城中村5个，住房保障工作被评为全国先进；海绵城市和地下综合管廊建设试点成效明显，获评国家装配式建筑示范城市。严格城市管理，大力开展人行道、渣土等专项整治，222个老旧社区和部分老旧公租房小区提质提档深入实施，湘江两岸和主干道亮化提质，城市更加清爽靓丽，蝉联全国文明城市。

　　五是着力促进城乡融合，建设全域美丽乡村。城乡基础设施加快对接，新建、改造农村公路744.7公里，完成水利工程建设3万余处、水毁工程修复8487处，改造危桥28座、农村危房9327户；优质公共服务加快向农村延伸，农村自来水普及率达85%，环境卫生整治、污水处理试点加快推进，农业废弃物资源化利用成为全国样板。都市型现代农业格局加快形成，新增土地合作社100家，培育省级以上农业产业化龙头企业79家，农产品加工销售收入突破2000亿元；培育创建一批特色小镇，乔口、灰汤新入选全国特色小镇，沩山获评全国美丽宜居小镇；加快创建美丽乡村示范村、特色村，梅田湖村成为全国生态文化村，休闲农业和乡村旅游经营收入达520亿元，“美丽经济”竞相发展。

　　六是着力强化基层基础，提升公共治理服务。社会治理与服务向网格延伸，推行“四位一体”网格管理，群众投诉减少一半以上。社会服务管理信息化“云”体系成型，公共资源交易信息实现“一网发布”，12345市民服务热线服务质量居全国第5位。基层治理持续加强，开展信访积案化解百日攻坚行动，一批信访积案有效化解；调解各类社会矛盾纠纷3.7万件，医患关系、交通事故、物业管理等纠纷调解体系基本建成；法律审查延伸到园区平台公司和重大民生项目，荣膺全国“法治政府建设典范城市”。平安长沙建设深入推进，安全生产事故、伤亡人数大幅下降，民族宗教领域和谐稳定，成为全国少数民族流动人口服务管理示范城市；禁毒力度不断加大，刑事案件持续减少，实现命案全破，涉恐涉爆案件消除在萌芽状态，成为全国首批“雪亮工程”示范城市，有力维护国家安全和公共安全；望城公安消防大队被国务院、中央军委授予“全国学雷锋模范消防大队”称号，浏阳市公安局获评“全国优秀公安局”，雨花区综合治理联勤联动模式在全国推介，长沙再获政法综治工作最高荣誉“长安杯”，获评“全国社会治安综合治理优秀城市”。

　　七是着力发展社会事业，增进人民群众福祉。十件民生实事全面完成。整治城区黑臭水体88处；新增公共停车场115个、停车位2.2万个；建成农贸市场（生鲜超市）食品安全快检室100个，改造“明厨亮灶”1.12万余家，食品安全检测合格率达99.7%，食品安全城市创建成效明显；贫困村义务教育标准化学校全面建成，配套幼儿园建设与移交加快推进，公办和普惠性幼儿园达75%，新增优质学位4万多个，解决教师编制6500个，芙蓉区率先实现公办普通高中免费入学，高等教育质量持续提升；城区每个街道至少拥有1家日间照料中心或小型养老机构，天心区荣获全国老龄健康能力服务示范城区，农村养老服务设施覆盖率达60%以上；农村建制村通客车率达99.7%；率先全国创新医疗卫生融资模式，城市公立医院综合改革全面推进，开通异地就医医疗费用直接结算，药品实现零差率销售，城乡居民大病保险、贫困人口基本医疗保险和残疾儿童康复全面实施；新改扩建乡镇卫生院（社区卫生服务中心）17所、建设标准化村卫生室70所；发放首批“城市一卡通”市民卡；完成80个住宅小区电力“专改公”，启动实施“电网供电能力提升三年行动计划”；不动产转移登记全面实行网签和资金监管；基本完成房产办证处遗工作。社会事业加快发展，梅溪湖国际文化艺术中心启用，炭河古城、秋收起义纪念园开园，湘剧《护国》走进国家大剧院，全市首台实景剧《浏阳河上》常态化演出，获评国家公共文化服务标准化示范地区。世界杯预选赛亚洲区中韩赛、国际马拉松赛、中华龙舟赛、市九运会等赛事成功举办。城镇新增就业14.2万人，城镇登记失业率控制在3%以内，城镇职工退休人员养老金、城乡居民基本养老保险基础金分别达到2304元、160元；城乡低保实现一体化，低保标准提高22%，特困供养标准提高228%；全体居民人均可支配收入预计达4.1万元，增长8.5%。长沙连续第十年获评中国最具幸福感城市。

　　一年来，各民主党派、工商联、无党派人士围绕中心、服务大局，参政议政、建言献策，推动了长沙发展。实行市领导领办民生建议和重点提案，办理人大代表建议305件、政协提案400件，办复率100%，满意率分别达96.5%、96.2%，有效维护群众利益。审计监督持续发力，审计整改机制进一步完善，促进增收节支17.75亿元。开展“小官大贪”专项整治，“雁过拔毛”式腐败问题立案649件，处分739人，政风行风持续好转。支持军队改革，国防动员、后备力量及人民防空建设持续加强。社科普及、科普服务、文艺服务走进基层群众，应急和机关事务管理有效推进，移民工作、职工维权积极开展，地方志、档案工作取得新成效。气象、水文、地震监测防灾减灾能力不断提升，慈善、福彩、红十字公益、抗洪救灾募捐彰显大爱。

　　一年来，我们突出发挥长沙在全省的示范引领、辐射服务作用，主动引领、对接长株潭一体化，城际交通、产业发展进一步深度融合；主动支援武陵山片区扶贫攻坚，投入2.07亿元全力助推龙山精准脱贫，区县（市）对口武陵山片区扶贫成效明显，以实际行动展示省会城市的担当与作为。

　　各位代表！过去一年成绩来之不易。这是省委省政府和市委正确领导的结果，是市人大及其常委会、市政协监督支持的结果，是全市上下团结奋斗的结果。在此，我代表市人民政府向全市各族人民，向各民主党派、工商联、无党派人士和人民团体，向驻长部队、武警官兵、政法干警和民兵预备役人员，向所有关心、支持和参与长沙发展的朋友们，表示诚挚的感谢和崇高的敬意！

　　同时，我们也清醒地看到，发展中还存在一些问题和不足：区域、城乡、群体之间发展不平衡问题比较突出，教育、医疗、食品安全管理、公共交通等公共服务供给不充分，居民收入增速与经济发展不同步；产业结构不优，优势产业、主导产业规模不大，与先进城市相比支撑发展的大项目、好项目不多，开放型经济、现代服务业等短板仍需补齐，外贸依存度明显偏低；大气、水等生态环境污染问题亟待解决，垃圾分类减量处理步伐不快；城市品质有待提高，城市建设管理不够精致精细，交通拥堵、停车难等“城市病”日益凸显；政府债务、房地产市场存在不同程度风险隐患；水利等基础设施不足，蓄滞防洪能力不强，安全保障标准不高；政务服务、营商环境不优，政府部门信息共享不畅，网上办事不能满足群众需求和企业需要，形式主义、官僚主义仍然存在，干部作风有待改进。对此，必须采取有效措施，切实加以解决。

　　二、2018年形势与目标

　　今年是贯彻党的十九大精神的开局之年，是改革开放40周年，是决胜全面建成小康社会、实施“十三五”规划承上启下的关键一年，做好今年的工作事关长远，至关重要。放眼全球，世界正处于大发展大变革大调整时期，面临的不稳定性不确定性突出；纵观国内，中国特色社会主义和经济发展进入新时代，社会主要矛盾已经转化，经济由高速增长阶段向高质量发展阶段转变，但仍处于并将长期处于社会主义初级阶段的基本国情没有变，经济长期向好的基本面没有变；审视长沙，我市发展来势向好、态势强劲，但仍处于转变发展方式、优化经济结构、转换增长动力的攻关期。新时代、新征程，再创业、再出发，我们必须以习近平新时代中国特色社会主义思想为指引，开拓进取，奋发有为，用实绩书写贯彻落实党的十九大精神的生动篇章。

　　新时代，必须瞄准新坐标、彰显省会新担当。长沙发展走在全国省会城市前列，已站上“万亿俱乐部”的新起点，步入高质量发展全面推进的新阶段，必须科学应对标兵渐远、追兵赶超的激烈竞争，抢抓新经济加快发展的“窗口期”，发挥国家级战略平台的叠加优势，对标世界优秀城市和国内发达城市，释放改革红利，打造创新高地、开放高地、制度高地、人才高地，抢占新一轮城市竞争制高点，建设彰显国际化品质、凸显长沙味道的现代化大都市。我们将在深度融入国家战略中彰显省会担当，充分发挥“一带一路”重要节点城市、长江经济带中心城市的优势，扎实推进长沙承担的国家改革试点，当好建设“一带一部”领头雁、落实“三个着力”先锋队，创建国家中心城市，加快打造国家智能制造中心、国家创新创意中心、国家交通物流中心，开启全面建设现代化长沙新征程。我们将在辐射、服务全省协同发展中彰显省会担当，发挥长株潭一体化核心作用，带动“3+5”城市群发展，助推武陵山片区扶贫攻坚，将长沙打造成为“五个强省”核心增长极、实施“创新引领、开放崛起”战略排头兵、建设“四大体系”“五大基地”示范引领区，提升长沙在建设富饶美丽幸福新湖南中的示范引领作用和综合服务能力。

　　新时代，必须把握新常态、践行发展新理念。新时代的发展，是追求更高质量、更有效率、更加公平、更可持续的内涵式发展。必须准确把握经济发展新常态这个大逻辑，将新发展理念贯穿于每项工作、每个环节，做到入脑入心，形成内生动力，成为行动自觉。必须坚持以质量效益为中心，推动经济发展质量变革、效率变革、动力变革，提高全要素生产率，形成创新成为第一动力、协调成为内生特点、绿色成为普遍形态、开放成为必由之路、共享成为根本目的的发展模式。特别是认真践行绿水青山就是金山银山的理念，像对待生命一样对待生态环境，像保护眼睛一样保护生态环境，实行最严格的生态环境保护制度，加强环境治理，促进绿色发展，建设人与自然和谐共生的美丽长沙。

　　新时代，必须直面新矛盾、实现群众新期盼。人民日益增长的美好生活需要和不平衡不充分的发展之间的矛盾是新时代的重要标志和重要特征，也是我们一切工作的出发点。必须始终坚持以人民为中心的发展思想，把人民对美好生活的向往作为奋斗目标，多谋民生之利、多解民生之忧，用心解决好交通拥堵、住房困难、环境污染和就业、上学、看病、养老等问题，一件接着一件办，一年接着一年干，切实满足群众更高要求的物质文化生活需要，不断满足群众对民主、法治、公平、正义、安全、环境等方面的要求，让长沙人民拥有更多获得感、幸福感、安全感。

　　今年政府工作指导思想是：全面贯彻党的十九大精神，以习近平新时代中国特色社会主义思想为指导，坚持稳中求进工作总基调，坚持新发展理念，紧扣我国社会主要矛盾变化，按照高质量发展的要求，统筹推进“五位一体”总体布局和协调推进“四个全面”战略布局，坚持以供给侧结构性改革为主线，深入实施“创新引领、开放崛起”战略，着力推进质量变革、效率变革、动力变革，加快建设国家智能制造中心、国家创新创意中心、国家交通物流中心，扎实做好稳增长、促改革、调结构、惠民生、防风险各项工作，打好“三大攻坚战”，加强和改善民生，促进经济社会持续健康发展，实现全面建设现代化长沙的良好开局。

　　全市经济社会发展主要预期目标是：地区生产总值增长8.5%；规模以上工业增加值增长8.5%；社会消费品零售总额增长10%；固定资产投资增长11.5%以上；一般公共预算收入增长10%；全体居民人均可支配收入增长8.5%；城镇登记失业率控制在4%以内；居民消费价格指数103左右；减排完成省定指标。

　　同时，按照高质量发展要求，更加注重以下指标：研发与试验发展（R&D）经费支出占地区生产总值比重2.6%以上；产业投资占固定资产投资总额比重35%以上；税收占财政收入比重72%以上；城镇保障性住房覆盖率23%以上；单位地区生产总值能耗下降3%以上。

　　实现上述目标，在工作中必须牢牢把握以下几个方面：

　　一是坚持稳中求进总基调，推动高质量发展。以习近平新时代中国特色社会主义经济思想为根本遵循，处理好“稳”和“进”的辩证统一关系，把握好工作节奏和力度，确保在经济增长、民生底线、风险防范上稳得住，在转换动能、调优结构、提高效益上进得好。坚持质量第一、效益优先，以供给侧结构性改革为主线，转变发展方式，推动实体经济、财政税收、群众生活、城乡建设、生态环境高质量发展。

　　二是把实体经济作为经济发展的着力点。坚持以先进制造业为引领，以工业园区为平台，以重大项目为抓手，围绕产业链部署创新链、强化人才链、完善政策链、保障资金链，构建实体经济、科技创新、现代金融、人力资源协同发展的产业体系。

　　三是着力抓重点、补短板、强弱项。将抓重点、补短板、强弱项作为干工作、谋发展的基本方法。强化问题导向，坚持底线思维，紧紧抓住解决不平衡不充分问题，加快推进产业转型升级，着力打好防范化解重大风险、精准脱贫、污染防治三大攻坚战，不断完善战略平台和重大基础设施，持续提升城市承载功能与品质，做强现代服务业、外向型经济，夯实行稳致远基础。

　　四是以政府转型带动发展全面转型。从转变政府职能入手，进一步明晰政府与市场的边界，充分发挥市场在资源配置中的决定性作用，让各种要素自由流动、各类市场主体活力迸发，更好发挥政府作用，推进简政放权、放管结合、优化服务，建设高效市场和有为政府，实现由审批型政府向法治政府、服务型政府转变，全面提升政府现代治理能力，推动经济社会全面转型。

　　五是锤炼重实干、强执行的作风政风。事业是干出来的。坚持“马上就办、办就办好”的原则，既雷厉风行又久久为功，以钉钉子精神做细做实做好工作，善于创造性推动实际工作，善于运用互联网和信息化手段开展工作，善于处理各种复杂矛盾和疑难问题，勇于攻坚克难，以强有力的执行力赢得政府公信力。

　　三、2018年主要工作

　　实现上述目标，我们将突出抓好以下十个方面的工作：

　　（一）突出做大做强实体经济。以智能制造为核心，树立产业链思维，强化要素保障，全面落实工业30条，提升先进制造业水平。狠抓产业链建设。以长株潭衡“中国制造2025”试点示范城市群建设为契机，突出22个工业新兴及优势产业链，加强产业横向集聚和垂直整合，聚焦中高端关键链节，打造竞争力强的先进制造业集群。巩固提升工程机械、汽车、电子信息、食品等优势产业链，加快实现新材料、人工智能及机器人、北斗导航应用、显示功能器件、增材制造、集成电路、智能电力装备、移动互联网等新兴产业链突破性发展，大力发展生物医药、检验检测、地理信息、基因工程等特色产业链，培育和引进“小巨人”企业、“独角兽”企业、行业领军企业。落实领导联点和产业链服务保障机制，实现工作力量、要素保障向产业链建设一线倾斜，形成更多千亿级产业集群，培育三千亿级、五千亿级产业集群。提升园区发展效益。坚持有所为有所不为，强化平台功能，聚焦主导产业，培育特色产业，疏解非核心产业，推动园区“腾笼换鸟”，实现更加集约、更有重点的发展。国家级园区瞄准五千亿级，迈向“大而强”，朝着主导产业鲜明、产业链条完整、产城深度融合的方向发展，打造现代工业经济综合体；省级园区瞄准千亿级，迈向“专而精”，朝着特色鲜明、要素集聚、配套完善的方向发展。加强园区公共服务平台建设，为产业和企业提供开放式服务。大力支持园区发展，推动优质资源向园区集中、优惠政策向园区倾斜，鼓励和支持有本事、想干事、会干事、能成事的干部到园区建功立业。加快工业互联网发展。深化“互联网+先进制造业”融合，系统推进工业互联网基础设施和数据资源管理体系建设，更好地发挥长沙工业云平台作用，整合线下专家、技术团队，实现供需精准对接、信息互动共享，推动制造业加速向数字化、网络化、智能化发展。新增智能制造示范企业100家，培育一批“中国制造2025”样板企业。开展“产业项目建设年”活动。坚持项目为王，深入推进“五个100”项目建设。力促广汽三菱发动机及整车技改、日写触摸传感器等项目建成投产；加快伟创力、中国通号二期、格力智能制造、柔性面板、隆平现代产业园等项目建设；启动磁浮装备产业园、智能终端产业园等项目建设，把一个个项目尽快转化为新的经济增长点。推行招商引资“一把手”负责制，深入开展产业链招商、二次招商，创新开展资本、院校招商，完善项目市级统筹机制，着力引进一批特大型产业项目，争取更多国家战略项目布点长沙。建设质量强市。进一步将质量和标准渗透到发展全过程，深化标准、质量、品牌一体化建设。申报创建全国质量强市示范城市，支持企业将核心技术转化为国家标准、国际标准，培育一批知名质量品牌。加强产业工人队伍建设，弘扬工匠精神，让更多“绝活”薪火相传。激发和保护企业家精神。始终把企业家作为创新和发展的中坚力量，加快建立快捷便利的准入机制，形成区域最低的商务成本，打造优质高效的政务环境，激发各类市场主体活力，倾力打造稳定公平透明、可预期的国际一流营商环境，让广大企业家放心投资、舒心创业、安心发展。依法保护民营企业的资产和权益，支持民营企业进行科技创新、管理创新，形成国有经济与民营经济比翼齐飞、双轮驱动的发展格局。

　　（二）突出以供给侧结构性改革为主线的各项改革。坚持以改革破难题、促发展，进一步提高改革精准性、系统性、有效性，将改革红利转化为发展活力。深化要素市场化配置改革。全面落实供给侧结构性改革“1+7”政策，深入推进“三去一降一补”。大力降低实体经济生产经营成本和制度性交易成本，继续清理涉企收费，实行涉企收费目录清单制度，研究制定降低企业用能、物流成本的政策措施，支持长沙高新区、望城经开区试点全国第二批增量购电改革。大力推进“放管服”改革。精准确定部门职能职责，持续推进简政放权，最大限度地减少行政许可事项。深化商事制度改革，继续清理后置审批事项，开展证照分离改革，破解企业“办照容易办证难”“准入不准营”等问题。深化“双随机、一公开”监管改革，构建及时、规范、高效的事中事后监管体系。优化审批服务，实施“多规合一”项目协同审批。强化园区管理体制改革。下放涉及工业企业投资项目的审批权限，申报试点园区相对集中行政许可权改革，实现“一个印章管审批、一张网络管服务”。设立市、园区两级企业服务机构，解决服务企业“最后一公里”难题。采取合并、托管等方式统筹整合园区，构建共建共管新模式。加快国资国企改革。围绕管资本为主，加快转变国有资产监管机构职能。完善分层分类管理体制，实现国资监管集中统一全覆盖；大力发展混合所有制经济，引进民间资本和战略投资者参与国企重组并购，推动国有资本做大做强做优。推进国有投资平台转型升级，降低资产负债率，完善现代企业制度，增强综合竞争力。启动公交企业等国企改革。协同推进其他改革。全力推进43项两型改革任务实现突破。支持监察体制改革。加快事业单位改革，逐步实现政事分开、事企分开、管办分离。改革教师管理体制，实现城区优秀教师跨区域无障碍流动，推动市属高校改革，扩大学校办学自主权。深化医药卫生体制改革，推进医联体建设。大力推进供销合作社综合改革，实施国库集中支付电子化改革，深化城区街道行政管理体制改革，加快律师、公证等改革。打好防范化解重大风险攻坚战。树立正确的政绩观，科学合理安排政府投资项目，创新融资方式，用好PPP模式；将政府债务纳入财政预算管理，清理融资平台债务，消化存量、控制增量，有效防范政府债务风险。坚决打击违法违规金融活动，守住不发生重大金融风险的底线。进一步完善房地产市场平稳健康发展长效机制，保持调控政策连续性、稳定性，防范房地产市场风险。

　　（三）突出建设创新开放高地。坚持在开放中创新、在创新中开放，使创新和开放真正成为引领长沙发展的核心动力。强化科技创新驱动。全面落实人才新政，加大人才引进力度，用好本土人才，保护尊重人才，充分释放人才红利。推进国家自主创新示范区建设，将政策覆盖到省级以上园区。加强国家重大科技专项对接，争取更多科研院所、国家重点实验室落户，发挥行业领军企业创新引领作用和智能制造研究总院等现有平台作用，建立以企业为主体、市场为导向、产学研用深度融合的协同创新生态体系，鼓励企业创建产学研创新联盟，开展重大产品创新，支持打造一批长沙智造、长沙创造优秀产品。强化知识产权创造、保护、运用，建成中国（长沙）知识产权保护中心，加快科技成果转移转化，推进宁乡经开区国家知识产权试点园区建设。落实科技创新政策，加大财政对科技的投入力度，设立科技创新基金，引导全社会加大科技研发投入。促进军民融合深度发展。以创建国家军民融合创新示范区为总揽，建立军民融合促进中心，加快“民参军”进程；创新军地融合机制，深化与国防科大等高校合作，组建军民融合先进技术研究院，促进优秀国防人才与技术“军转民”。推进湖南省军民融合科技创新产业园、长沙军民融合双创示范园建设。加强开放平台建设。支持开通长沙至北美、欧洲等国际全货机航线，申报一类铁路口岸，拓展“五定”班列（轮）新路线，促进中欧班列、湘粤港直通快车持续稳定运营；高效运行黄花综合保税区。提高通关效率，优化出口退税、口岸清关监管流程，拓展粤港海关通关模式，实现通关一体化。推进国际化进程。加强国际产能合作，推动优势产能“走出去”，大力发展加工、转口和新型贸易，提升经济外向度。发展国际友好城市，积极引入国际政商机构，完善国际化标识系统，承办首届中国国际商事法律服务高峰论坛，推进重点领域公共服务国际化，建设一批国际学校、社区和医疗机构，让长沙更有“国际范”。引领长株潭城市群发展。强化省会担当，突出打通市际断头路，推进产业发展、基础设施、公共服务和环境治理等全方位对接，在交通物流、科技创新上率先融合，形成大都市城市群格局。抱团深度融入“一带一路”建设，加强与长江中游城市群、粤港澳大湾区等地区城市合作；加快金阳新城建设，推进湘赣边区域合作经济带建设。

　　（四）突出增进群众获得感。始终把人民利益摆在至高无上的地位，强化民生优先，重点突出宜居，让老百姓的获得感、幸福感、安全感在家门口升级。让老百姓呼吸更新鲜的空气、喝上更干净的水。坚决打好污染防治攻坚战，重点打赢蓝天保卫战。加强大气污染源头控制，鼓励使用清洁能源汽车，大力推广新能源公交车；严控农村秸秆焚烧，深化粘土砖厂、燃煤锅炉整治，完成老旧社区餐厨油烟净化8万户；强化建设施工现场扬尘防治，实现主城区扬尘在线监测全覆盖，确保空气质量优良率持续提升。围绕湘江保护和治理“一号重点工程”，全面落实“河长制”改革措施，探索“湖长制”“山长制”，加强流域综合治理。深入实施“水十条”，加强湘江干流长沙段和株树桥水库等饮用水源保护，新建七水厂、扩建望城水厂、提质改造一水厂，加快雷锋水质净化厂建设；加强住宅供水改造和设施管护，提高二次供水质量；加大农村养殖污染治理力度，巩固提升农村自来水普及水平；加快敢胜垸、苏圫垸等污水处理厂建设和新开铺污水处理厂扩容、花桥污水处理厂提标，推进浏阳河下游污水收集，提高污水处理出水排放标准。让老百姓吃得更放心。抓好“菜篮子”工程，实施优质粮食工程。推进餐饮店“透明厨房”建设，建成一批早夜市示范点，规范建设一批便民新鲜农产品超市。进一步强化食品安全监管，提升农产品检验检测能力，基本实现食品安全追溯体系全覆盖，确保农产品质量安全，建成国家食品安全示范城市。让老百姓出行更便捷。坚持公交优先、慢行优先，大力发展公共、轨道交通，优化公交线路，营造良好出行环境。打通一批瓶颈路、断头路，加强人行道专项整治，建成自行车道320公里，优化城区路网体系。实现中心城区公交站点500米覆盖率98%以上，建成国家公交都市示范城市。让老百姓住得更有保障。坚持“房子是用来住的、不是用来炒的”定位，加快建立多主体供应、多渠道保障、租购并举的住房制度。加大城中村改造力度，实施棚改新三年行动计划，加快在建商品房建设，启动一批定向限价商品房和园区人才公寓建设。发展住房租赁市场特别是长期租赁，支持专业化、机构化住房租赁企业发展，发挥好住房公积金作用，打赢房价保卫战。引导和支持老旧居民楼加装电梯。让老百姓享受更优质的公共服务。推动城乡义务教育一体化发展，建设义务教育标准化学校300所，基本完成小区配套幼儿园移交。提质提档市级医院，建设市中心医院综合楼、一医院新住院大楼、四医院滨水新城院区和残疾人康复中心，启动市中医院医养大楼建设，筹建市妇幼医院分院、口腔医院分院；加强基层医卫人员培训，新建乡镇（街道）卫生院6个、村（社区）卫生室30个。加强县（市、区）和乡镇（街道）公共法律服务实体平台建设，实现村（居）法律顾问全覆盖。让老百姓获得更广泛更实惠的民生福祉。坚持就业优先，促进高校毕业生等青年群体、农民工多渠道就业创业，援助残疾人、低保对象等困难群体就业，城镇新增就业13万人，零就业家庭100%动态清零。织密织牢社会保障网，实施全民参保计划，启动长期护理保险试点，实现城乡居民养老保险基础养老金“12连调”，失能人员长期护理费用报销比例达70%，城乡居民医疗保险特殊病种增至43种、普通门诊统筹报销比例提高10个百分点。关爱老人，实施高龄津贴扩面提标，建成40个乡镇敬老院照料护理区。加强劳动保障监察执法，切实维护劳动者合法权益。让老百姓生命财产更安全。深化平安长沙建设，建立信访积案化解长效机制，健全立体化信息化社会治安防控体系，提高打击新型犯罪能力和防恐处突水平，加强禁毒工作。强化学校、幼儿园安全工作，决不让孩子受到侵害。提升防灾救灾减灾和应急能力，实现气象预警全网发布，加强城镇防洪保护圈建设，实施黄材等大中型水库水闸以及堤坝除险加固工程。加快推进12个中心消防站和11个消防应急取水设施建设。落实企业主体责任，抓好重点领域和行业隐患排查整治，坚决遏制重特大安全事故发生。办好十件民生实事。一是完成棚户区改造11461户，改造农村危房3560户。二是新建停车场100个，新增停车位2万个。三是提质改造农村公路400公里。四是新改扩建公办幼儿园30所。五是创建垃圾分类减量示范小区20个。六是创建绿色示范村庄、示范社区100个。七是做好4.6万名适龄妇女两癌筛查和5万名孕产妇产前免费检查。八是建设标准化母婴室10个。九是为500名0-14岁残疾儿童提供抢救性康复救助。十是新建全民健身工程600处。

　　（五）突出建设战略平台和重大基础设施。始终贯彻新发展理念，坚持高起点高标准规划建设，着力打造彰显长沙形象的窗口、引领未来发展的标杆。加快湖南湘江新区发展。围绕“三区一高地”战略定位，发挥龙头作用，走出一条创新引领、产业支撑、统筹融合的经济功能区转型发展路子，打造湖南增长极和长株潭城市群发展的排头兵。突出提升新区功能，更加注重实体经济发展、产业转型升级，布局人工智能、生命基因等未来产业，统筹新区各园区重大产业布局。抓好岳麓山国家大学科技城、湖南金融中心、大王山、长沙高铁西城、沩东新城等重点片区建设，加快梅溪湖国际新城二期、月亮岛文旅新城建设，提质洋湖片区。完善综合交通规划，推进麓景路南延、长望路西延建设，打通西二环交通堵点。拉通潇湘大道、含浦大道。加快国家双创示范基地、军民融合创新示范基地建设。抓好基因谷、亿达制造小镇、宜家荟聚等重大项目，促进高端制造和高端服务业快速集聚。深化新区体制改革，理顺执法体制，统筹规划交通产业服务。推进重大片区建设。岳麓山国家大学科技城以建设全国领先的自主创新策源地、科技成果转化地和高端人才集聚地为目标，实施品质提升、合作共创工程，重点提质改造麓山南路等区域，优化功能布局；构建“基金+基地”创投孵化新体系，打造产业小镇，扶持“两山一湖”、后湖、西湖文化园文创产业发展。马栏山视频文创产业园瞄准“北有中关村、南有马栏山”的目标，全力打造具有国际影响力的“中国V谷”，加快湖南广电产业落地建设，创新政策举措，吸引更多企业、团队和人才入驻。临空经济示范区围绕打造“中部地区内陆开放高地”，重点抓好自贸区改革试点经验复制推广，编制总体规划，加快建设国际航空和空铁联运枢纽，引进和发展航空物流、航空服务等航空偏好型产业。高铁会展新城围绕打造国际中高端会展和会议承办地、现代服务业聚集新区，紧扣会展全周期产业链，完善片区规划设计，推进国际会议中心和高铁商务区建设。湖南金融中心围绕建设区域金融中心、特色金融示范区，重点完善交通规划和城市设计，推动金融机构、要素平台、中介机构集聚，争创国家级金融综合改革创新试验区。南部片区围绕建设长株潭融城核心区、城市群生态绿心样板区、全国两型发展示范区，重点完善规划修编，加强“绿心”保护，推进工业企业全面退出，加快长株潭商圈建设，大力发展现代商贸、生态旅游、文化、康养等高端服务业。完善重大基础设施。启动黄花机场T3航站楼建设。推进渝长厦高铁长益常段、蒙华铁路建设，实施高铁长沙西站工程，加快长沙至浏阳、宁乡轨道交通建设前期工作。推进长沙至益阳高速复线、江背至干杉、宁乡至韶山高速建设，建成岳望高速。加快地铁3、5、6号线工程建设，启动1号线北延一期工程，实现4号线试运行。优先建设过江通道，启动湘雅路、兴联路等过江通道建设。推进湘府路快速化、107国道暮云段、潇湘北路、中青路北延线改造和湘江大道南延线、时代阳光大道东延线建设，加快汽车南站综合交通枢纽、国际铁路港、长沙新港建设。推进白石洞水库建设，启动椒花水库枢纽工程前期工作。实施电网供电能力提升三年行动、“630攻坚”计划。启动黑麋峰生活垃圾焚烧二期建设，开展河东、河西生活垃圾清洁焚烧项目选址及前期工作。

　　（六）突出实施乡村振兴战略。按照产业兴旺、生态宜居、乡风文明、治理有效、生活富裕的总要求，加速农业农村现代化，让农业成为有奔头的产业，让农民成为有吸引力的职业，让农村成为安居乐业的美丽家园。促进乡村经济繁荣。完善城乡融合发展体制机制，清除要素下乡障碍。推进强镇扩权，形成镇域经济“聚合效应”；深化“三权分置”、集体经营性资产股份合作制改革，盘活集体资源资产，保障农民财产权益，壮大集体经济，激发乡村振兴内在活力。坚持质量兴农、绿色兴农，大力发展现代都市农业，健全现代农业产业体系、生产体系和经营体系。实施农业品牌建设五年行动计划，培植壮大农产品加工龙头企业和全国知名品牌。加强耕地保护与轮作休耕，完成30万亩农作物种植结构调整，打造30个千亩、4个万亩特色种植片区。加快现代农业产业园建设，促进一二三产业融合发展。推进农业科技创新和成果应用，增强农业综合生产与抗风险能力。鼓励发展多种形式适度规模经营，加快培育新型职业农民和现代农业经营主体。打造美丽乡村升级版。加强乡村规划，优化功能分区，建设100个市级美丽乡村示范村、100个特色村，创建一批全域美丽乡村示范乡镇、示范县。全面修复农村水毁工程，补齐农村公共服务、信息网络等短板。实施农村人居环境整治三年行动计划，探索农村公共环境卫生管理、污水治理新机制，大力发展畜禽生态环保养殖，加快乡镇污水处理厂建设和常态化运营。加强乡村文明建设。坚决打赢脱贫攻坚战。开展已脱贫对象“回头看、回头查、回头帮”，建立健全稳定脱贫长效机制，巩固脱贫成果，提高脱贫质量。落实好对口帮扶精准脱贫攻坚专项行动计划，在全省脱贫攻坚中彰显省会担当。

　　（七）突出发展现代服务业。以先进制造业为基础，补齐现代服务业短板，加快消费升级，培育新的经济增长点。加快发展现代金融。建立企业上市挂牌联席会议和绿色通道制度，实施境内和境外上市并重策略，进一步加大企业上市力度；引进培育一批并购基金和中介服务机构，发挥好政府产业引导母基金的作用，加大引进合作力度，推进湘江基金小镇建设；加快长沙银行上市，引导长沙农商银行业务转型，做大做强本土法人银行；鼓励保险公司通过投资股权、债权、基金、资产支持计划等多种形式，为基础设施建设和战略性新兴产业发展提供资金支持；创新科技金融、绿色金融和互联网金融发展，增强金融服务实体经济能力；组建地方资产管理公司、农业保险公司、金融租赁公司和政府性融担公司，争取更多金融牌照，逐步构建全牌照法人金融机构体系。加快发展现代物流。推进供应链体系建设，提高供应链协同效率；推广物流标准化，支持发展电商物流、保税物流、冷链物流和农村物流，积极发展多式联运；续建海吉星农产品物流园和红星农副产品全球采购中心，建成恒广智能仓储配送中心、传化公路港。加快发展电子商务。依托互联网平台，大力发展共享经济。积极引进电商企业，申报建设国家跨境电子商务综合试验区，支持跨境电商等新业态发展，扶持发展农村电商。加快发展全域旅游。围绕建设世界旅游目的地，推进“旅游+”产业发展，实施旅游公共服务体系建设新三年行动计划，持续推进“厕所革命”，支持望城区、长沙县、浏阳市、宁乡市创建国家全域旅游示范区，支持耀邦故里、开慧故里创建国家5A级景区，建好华强文产园、新华联铜官窑、书堂山国学小镇等新旅游景点，推进浏阳河文旅产业带建设。加快发展楼宇经济。把发展总部经济与培育特色优势产业集群相结合，吸引总部企业聚集发展；优化楼宇核心功能，形成一批科技服务、工业设计、服务外包特色楼宇，打造“立起来的园区”。加快发展会展经济。围绕培育千亿会展产业、壮大特色支柱产业，办好工程机械、轨道交通、创新创意等特色会展，引进有国际影响力的展会落户，举办中国（长沙）智能制造峰会、中国国际物流发展大会、中国国际农产品交易会、全国糖酒会，建设会展名城。

　　（八）突出打造美丽长沙。坚持以人为本、优化布局、生态文明、传承文化的原则，加快建设和谐宜居、富有活力、独具特色的现代化大都市。坚持规划引领。树立精明增长、紧凑城市理念，科学编制城市总体规划、远景战略规划，确定城市人口规模，划定“三区三线”，实施全域规划，落实“多规合一”，严格刚性管控，构建城市理想空间格局；深度完善城市设计，以湘江为轴优化交通轴、产业轴、文化轴、生态轴和景观轴，精心设计街区、街景，让街巷更有人情味；优化综合交通体系规划，系统解决交通拥堵等“城市病”。凸显文化灵魂。坚持以文铸城，放大“古色”湖湘文化、“红色”革命文化、“绿色”生态文化特色，吸收外来先进文化，擦亮山水洲城厚重文化名片，增强文化凝聚力和城市软实力。加大历史遗存和文化名镇、名村保护，加快历史步道、文化街区建设，加强湘江古镇群保护与开发。提升建设品质。着力打造百年品质工程，使城市建设的每一个单元、每一个细节都精致精美。引导建设一批地标性建筑，鼓励争创鲁班奖，大力推广绿色、装配式建筑。有序实施城市更新，推进红星等15个城市片区“双修”，建设海绵城市，加快圭塘河生态修复。开展综合管廊试点和建设，加强城市地下管线工程档案保护。规范城市建设形态和色彩，严控湘江两岸建筑，打造沿江景观带，充分展现长沙独特风貌。提升管理品质。完善以网格为基本单元的管理模式，让老百姓的事情不出网格就能办结。推进城市管理执法改革，强化职责权限划分。推行垃圾分类减量处理，实行路面、立面、水面全覆盖保洁，着力整治车辆乱停乱放、违规户外广告招牌等乱象，加强共享单车管理，精心建设一批市容严管示范路段，全面提高市民素养和社会文明程度，建设更高水准的全国文明城市。提升生态品质。开展高质量的国土绿化行动，实施退耕还林还湿还草400公顷，新增城区绿地360公顷，建设洞井体育公园，建成开放狮子山、海棠公园和马楚文化园，让街心转角、街巷庭院、立面空地处处充满公园气息。实施河湖连通工程，启动创建国际湿地城市、国家生态园林城市，实现城市建设与生态环境完美融合，让好山好水遍布长沙大地。提升社区品质。改造社区环境，完善服务功能，规范和提升小区物业管理服务，完成50个社区、50条支路街巷提质改造；建设一批都市园艺社区、特色风貌街巷；构建“15分钟社区生活圈”，全面提升老百姓家门口的城市品质。

　　（九）突出发展社会事业。坚持社会事业与产业协调、均衡发展，推动公共资源向农村覆盖、向基层倾斜。建设教育强市。推动优质教育资源均衡发展，着重解决乡村教师队伍结构短缺问题。办好特殊教育、网络教育和普惠制学前教育，支持并规范民办教育发展，整治教育培训市场，着力解决中小学生课外负担重、“择校热”“大班额”等突出问题，解决好婴幼儿照护和儿童早期教育服务问题。完善职业教育和培训体系，筹建长沙幼儿师范高等专科学校，建成长沙卫生职业学院新校区，启动湖南信息职院新校区建设。支持高校“双一流”建设。打造健康长沙。推进智慧医疗建设，完善医疗、医保、医院、医药综合配套。加快“健康长沙”PPP项目建设，提高基层医疗服务水平，加强慢性病防治，健全疾病防控、应急救援、妇幼保健、血液管理等公共卫生服务体系和平台。发展老龄事业和产业，鼓励社会资本进入，统筹推进居家养老、社区养老和机构养老，促进医养健康产业发展。传承发展中医药事业，创建全国基层中医药工作先进市。发展体育事业。大力发展社区体育，完善公共体育设施，广泛开展全民健身运动，创建国家运动健康城市。鼓励社会资本投资体育领域，争取更多体育赛事落户长沙，培育马拉松、青少年足球赛等本土赛事，筹办中日韩青少年运动会，以运动赛事带动竞技体育和体育产业发展。加快国际体育新城、千龙湖休闲体育小镇建设。促进文化繁荣。坚持以文惠民，实施乡村文化振兴工程，推动公共文化“进村入户”，加强职工文化阵地建设，发展文艺事业，推出精品力作，推进全民艺术普及工程，开展媒体艺术之都系列活动，建设“书香长沙”。推进“文化+旅游、消费、科技、金融”深度融合，做强做优影视传媒、动漫游戏、数字创意、广告等文创产业；加快国家级文化科技融合示范基地、国家文化消费试点城市建设。推动其他事业发展。筹备纪念刘少奇同志诞辰120周年活动。深化“七五”普法。开展全国第四次经济普查、第三次土地调查。深入推进国防动员和民兵预备役调整改革，加强全民国防教育，维护军人合法权益，提升双拥工作水平。促进民族团结进步，加强宗教服务管理。培育优秀青年岗位能手，发展妇女、儿童事业，提升慈善、红十字公益服务水平，增强社会科学服务发展能力。编修长沙方志，规范档案管理。推进“侨梦苑”、海峡两岸青年创业园建设，做好移民工作，促进各项社会事业蓬勃发展。

　　（十）突出加强政府建设。始终坚持党的领导，牢固树立 “四个意识”，提高政治站位，转变政府职能，提升政府治理现代化水平，增强政府公信力和执行力。打造服务型政府。把群众和企业满意作为政府服务的第一标准，优化工作流程、提升办事效率。深化“互联网＋政务服务”，加强行政审批服务规范化、标准化建设，让群众和企业到政府办事基本实现“最多跑一次”。优化便民服务，将部门热线整合至12345市民服务热线。推动政策落地标准化管理，形成政策落地“长沙标准”。提高公务人员学习能力，以学习力提升创新力、执行力。打造智慧政府。实施数据驱动，把云计算、大数据、互联网作为优化政府服务的基础设施和重要支撑，突出电子政务数据信息交换平台和共享平台建设，建成智慧政务云，打造“城市数据大脑”。加强政企合作、多方参与，打通信息壁垒，推动部门数据无条件归集、有条件使用，实现技术、业务、数据融合，深度开发便民应用，让百姓少跑腿、数据多跑路，促进政府决策科学化、社会治理精准化、公共服务高效化。打造法治政府。健全法律框架体系，落实党政主要负责人的法治职责，深入推进依法行政。主动接受人大及其常委会的法律监督、工作监督，办好人大代表建议、意见，积极回应人大执法检查和专题询问。自觉接受政协和各民主党派、工商联、无党派人士的民主监督，办好政协提案。用好立法权，加强关键领域、关键行业立法，坚持公正文明执法，加强行政合法性审查，推动重大投资项目法律审查全覆盖，防范重大行政决策法律风险，促进政府管理规范化，用法治点亮城市之光。打造廉洁政府。开展“不忘初心、牢记使命”主题教育活动，严明政治纪律和政治规矩，严格落实中央八项规定精神，进一步纠正“四风”，以永远在路上的恒心和韧劲抓好作风建设，落实党风廉政建设主体责任和“一岗双责”，标本兼治遏制腐败和不正之风。强化财政预算管理约束力，提高公共资金使用效率，集中财力办大事、办实事。深入推进审计监督，加大公共资金、国有资产、国有资源监管力度，规范公共资源交易和招投标，把制度的笼子扎得更牢。

　　各位代表，新时代必须有新担当，奋发作为方能不负重托。让我们紧密团结在以习近平同志为核心的党中央周围，在省委、省政府和市委的坚强领导下，满怀激情开启新征程，勤勉实干谋求新作为，锐意进取，开拓拼搏，奋力谱写全面建设现代化长沙的新篇章。
